# Spatial pattern of soil organic carbon and total nitrogen, and analysis of related factors in an agro-pastoral zone in Northern China

**DOI:** 10.1371/journal.pone.0197451

**Published:** 2018-05-17

**Authors:** Xuyang Wang, Yuqiang Li, Yinping Chen, Jie Lian, Yongqing Luo, Yayi Niu, Xiangwen Gong

**Affiliations:** 1 Northwest Institute of Eco-Environment and Resources, Chinese Academy of Sciences, Lanzhou, China; 2 University of Chinese Academy of Sciences, Beijing, China; 3 Naiman Desertification Research Station, Northwest Institute of Eco-Environment and Resources, Chinese Academy of Sciences, Tongliao, China; 4 School of Environmental and Municipal Engineering, Lanzhou Jiaotong University, Lanzhou, China; Beijing Normal University, CHINA

## Abstract

The spatial pattern of soil organic carbon (SOC) and total nitrogen (TN) densities plays a profound important role in estimating carbon and nitrogen budgets. Naiman Banner located in northern China was chosen as research site, a total of 332 soil samples were taken in a depth of 100 cm from the low hilly land in the southern part, sandy land in the middle part and an alluvial plain in the northern part of the county. The results showed that SOC and TN density initially decreased and then increased from the north to the south, The highest densities, were generally in the south, with the lowest generally in the middle part. The SOC and TN densities in cropland were significantly greater than those in woodland and grassland in the alluvial plains and for Naiman as a whole. The woodland SOC and TN density were higher than those of grassland in the low hilly land, and higher densities of SOC and TN in grassland than woodland in the sandy land and low hilly land. There were significant differences in SOC and TN densities among the five soil types of Cambisols, Arenosols, Gleysols, Argosols, and Kastanozems. In addition, SOC and TN contents generally decreased with increasing soil depth, but increased below a depth of 40 cm in the Cambisols and became roughly constant at this depth in the Kastanozems. There is considerable potential to sequester carbon and nitrogen in the soil via the conversion of degraded sandy land into woodland and grassland in alluvial plain, and more grassland should be established in sandy land and low hilly land.

## Introduction

Soil is a huge C pool and plays an important role in global warming due to greenhouse gas emission and mutual impact on nitrogen cycle. For example, soil emission of carbon dioxide into the atmosphere is estimated to be six times the amount derived from fossil fuels [1, 2]. The global soil carbon pool (2500 Gt) is three times the size of the atmospheric carbon pool (760 Gt), 4.5 times of the biotic pool (560 Gt) [3]. In addition, restored SOC and TN are important for restraining land degradation [4, 5], because they create soil conditions that sustain vegetation and reduce the risk of land degradation.

Although drylands store less SOC per unit area than that in humid regions, the gigantic area covered by the world’s dry-lands (nearly 40% of the total land area) makes them a crucial global carbon sink; indeed, the potential SOC stock per unit area in dry land may be comparable to that in soils of the humid areas [6]. A large dry-land soil sink capacity would develop when large amounts of SOC are lost through degradation and can be replaced by ecosystem restoration efforts, which is not necessarily the case for soils in humid regions [7]. Desertification leads to losses of large amounts of carbon from the plant–soil continuum, particularly when the degradation results from unsustainable human activities and climate change [8]. In such situations, soil carbon sequestration and stocks could be improved by implementing appropriate land use practices [9, 10]. Thus, between different land uses, SOC and TN differ greatly due to the distinct soil management practices and plant covers [11]. In addition, between different soil types, soil nutrients have great impact on soil organic matter (SOM) input via vegetation productivity [12], on the other hand, SOM losses will be significantly reduced through the role of clay in inhibition of decomposition [13]. Therefore, it is essential to investigate SOC and TN changes under different land uses and soil types.

Horqin Sandy Land located in the eastern part of Inner-Mongolia of China, once covered by lush vegetation dominated by palatable grass species, and it was an important pastoral region of Inner Mongolia before 1950. However, as result of a population boom and widespread livestock grazing, the region’s landscape began to undergo severe desertification in the 1950s [14]. The desertification has been aggravated by extensive cultivation of unsuitable land, unsustainable firewood harvesting, and excessive groundwater withdrawal, leading to dominance of the region’s landscape became dominated by areas of active sand dunes since the 1970s, with a very low vegetation cover (less than 5%) [15]. In our previous research, we studied the rate of accumulation of carbon and nitrogen in the plant–soil system after restoration of active sand dunes [16], SOC and TN storage under different land uses were not studied for the whole Naiman Banner, where located in Inner Mongolia Autonomous Region of China. In the present study, our goal was to obtain this missing information.

The spatial pattern of SOC and TN densities are influenced by the distribution of soil types and land uses, as well as by the topography [17]. Due to the diversity of factors, it may exhibit a strong spatial heterogeneity of SOC and TN in both the horizontal and vertical directions [18]. Thus, incorporating and understanding such heterogeneity and spatial pattern characteristics can improve the precision of regional carbon and nitrogen budgets, thereby assisting in the development of effective ecological restoration measures. There have been considerable research about the spatial variability of SOC and TN in different regions by using geostatistics, which is based on the theory “regionalized variable”, and the results show that SOC and TN exhibited non-uniform spatial distribution mainly due to the high variability in soil parent material, land use/cover and geological factors [19–24]. However, there has been no quantitative research on this spatial variability in sandy grassland, where the fragile ecological environment and frequent human activities create ecosystems that are more sensitive to their environment and to global climate change than is the case for most other ecosystems. Thus, it is necessary to study the spatial variability of SOC and TN using tools such as GIS and geostatistics in areas such as China’s Naiman Banner, which has become an important part of northern China’s semiarid agro-pastoral ecotone.

The objectives of this study were to (1) describe the spatial variation of SOC and TN densities in this region, (2) reveal the causes of differences in SOC and TN densities by comparing the density among the region’s main soil types and land use patterns, and (3) support the development of a management strategy for land use and ecological restoration. The results of this research will significantly improve our understanding of the spatial distribution of SOC and TN densities and the underlying causes in this ecologically fragile region.

## Materials and methods

### Study area

**Fig 1:**
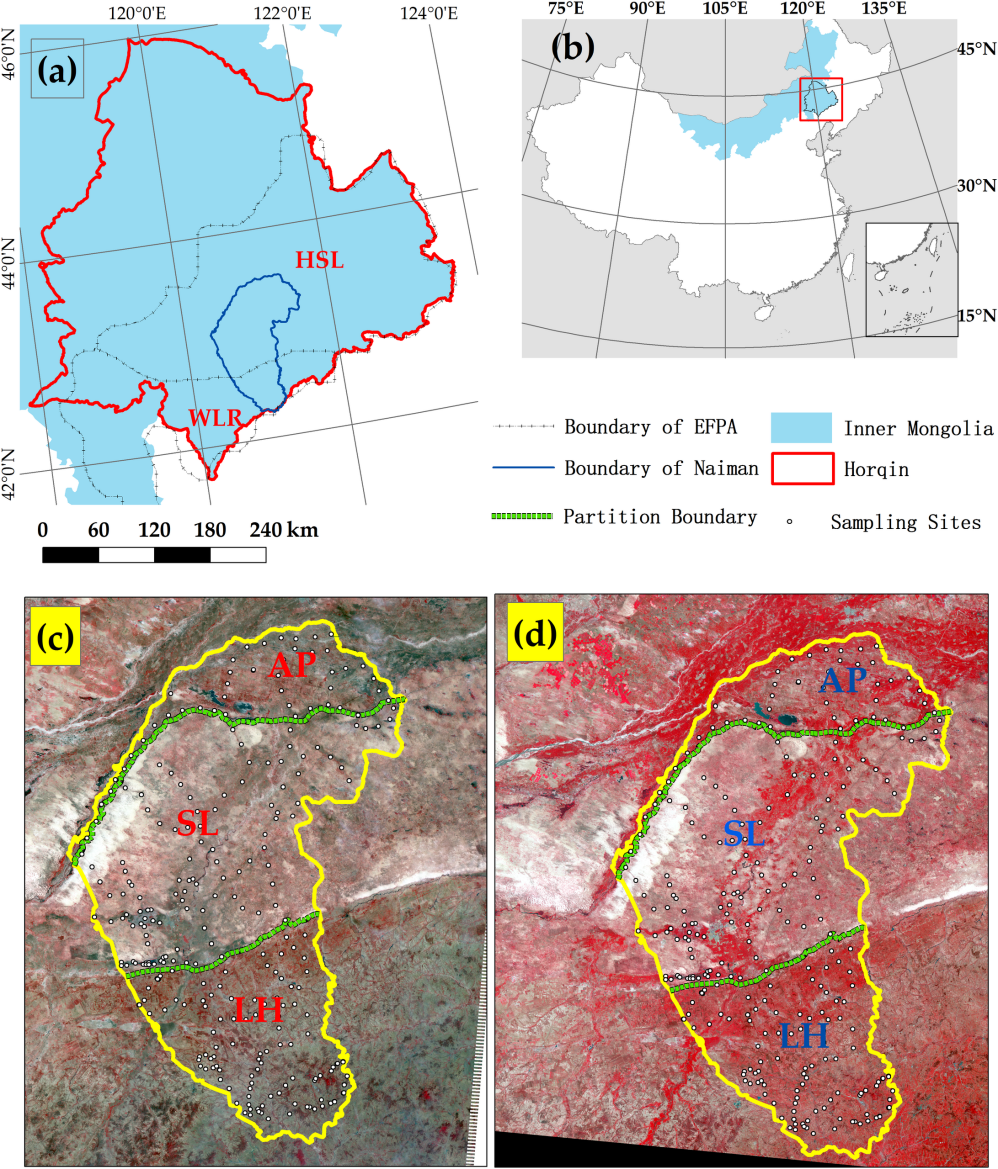
Location of the study area and sampling sites. **Reprinted from [27] under a CC BY license, with permission from [Jie LIAN], original copyright [2017].** (a) Naiman Banner is located in the southern part of the Horqin Sandy Land (HSL). The northern and central areas of Naiman Banner belong to the national ecological function protected area (EFPA); the southern part belongs to the Source Region of the West Liaohe River (WLR). (b) The research area is located in the southeastern part of Inner Mongolia, in northern China. The region has been divided into three zones (AP, alluvial plains; SL, sandy land; LH, low hills) based on the soil and land use characteristics and Landsat ETM+ imagery from (c) June 2010 and (d) July 2015.

Our study was conducted in Naiman Banner, which covers an area of 8137.6 km^2^ in the southern part of the Horqin Sandy Land of China’s Inner Mongolia autonomous region (Fig 1A and 1B). This region includes a wide variety of landscape types due to the large variation in environmental and land use characteristics, combined with extensive restoration and management practices in the past several decades to combat widespread desertification. The desertified area accounted for 69.5% of the total area of Naiman Banner by the late 1970s, when the region’s landscape became dominated by mobile dunes [25]. Since then, many fixation projects have been implemented in the region, primarily by means of the establishment of grazing exclosures to protect the remaining grassland and of afforestation using indigenous and introduced shrub and tree species to stabilize and protect soils. The extensive restoration practices have significantly reversed desertification, primarily due to the conversion of sandy land into woodland and restored grassland.

Naiman Banner ranges in elevation from 226.6 m asl in the northeast to 794.5 m in the southwest. The northern and central areas of Naiman Banner lie in a national “ecological function protected area” (an area that is formally protected to ensure that it can sustainably provide ecosystem services) within the Horqin Sandy Land, where the land’s functions include provision of a windbreak and sand fixation. The southern part belongs to the Source Region of the West Liaohe River protected area, an area mainly to water conservation (Fig 1A). We divided the Horqin Sandy Land into three parts based on the land characteristics detected in Landsat ETM+ satellite images with a resolution of 30 × 30m from June 2010 and July 2015 (Fig 1C and 1D): the northern part of the region is dominated by alluvial plains, the central part is characterized by sandy land dominated by sand dunes that alternate with gently undulating interdunal lowlands, and the southern part is dominated by low hills. This region is a typical desertified area, and as such, the Chinese Academy of Sciences established the Naiman Desertification Research Station (42°55'52"N, 120°41'56"E, 377 m asl) in 1985 to support research in this region, and has been conducting ecological restoration research at the station ever since [26].

**Fig 2:**
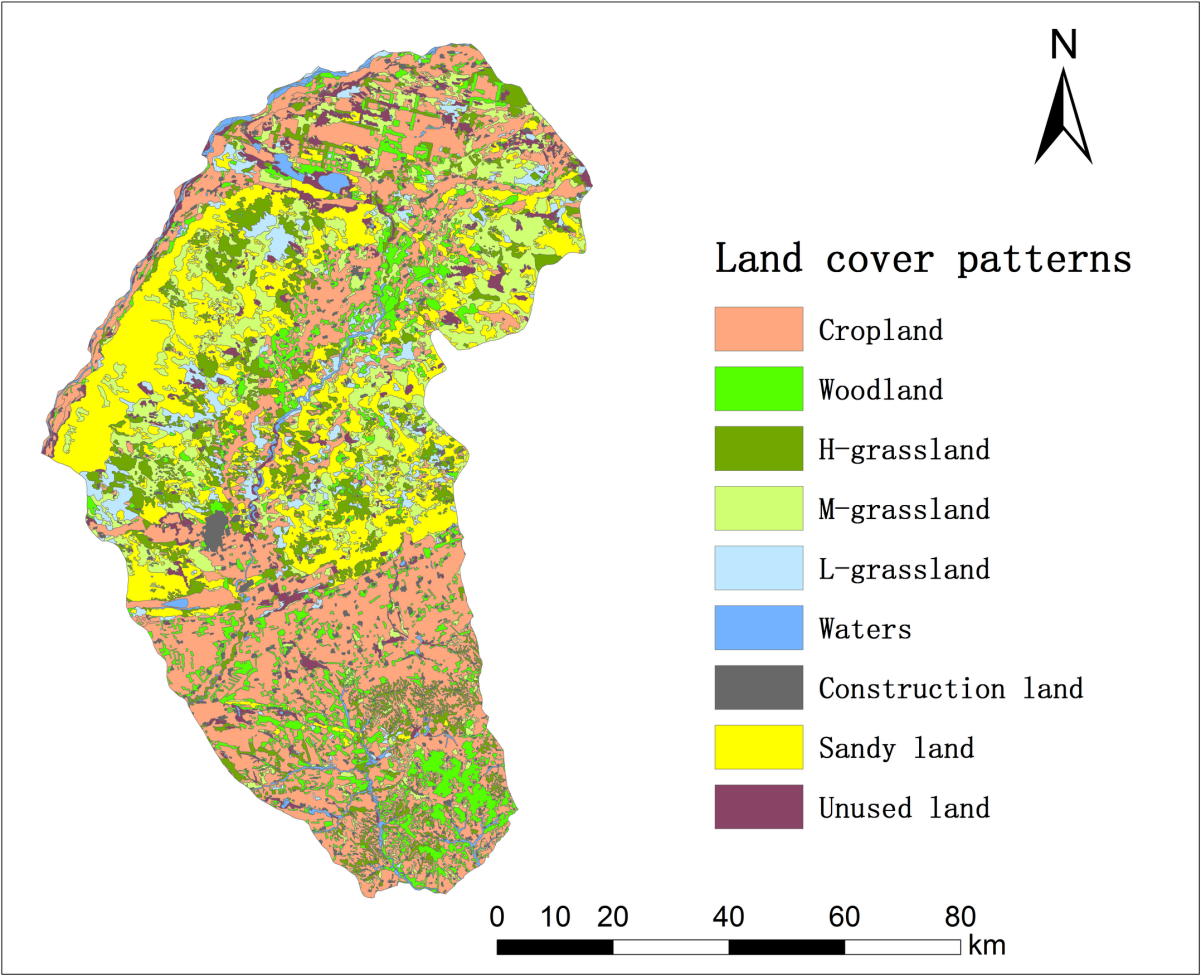
Land use pattern in Naiman Banner in 2015. **Reprinted from [29] under a CC BY license, with permission from [Xu Xinliang], original copyright [2016].** Three types of grassland were defined: H, M, and L, which represent high (> 50%), medium (> 20–50%), and low (> 5–20%) vegetation cover, respectively. The dataset was provided by the Data Center for Resources and Environmental Sciences, Chinese Academy of Sciences (http://www.resdc.cn).

**Fig 3:**
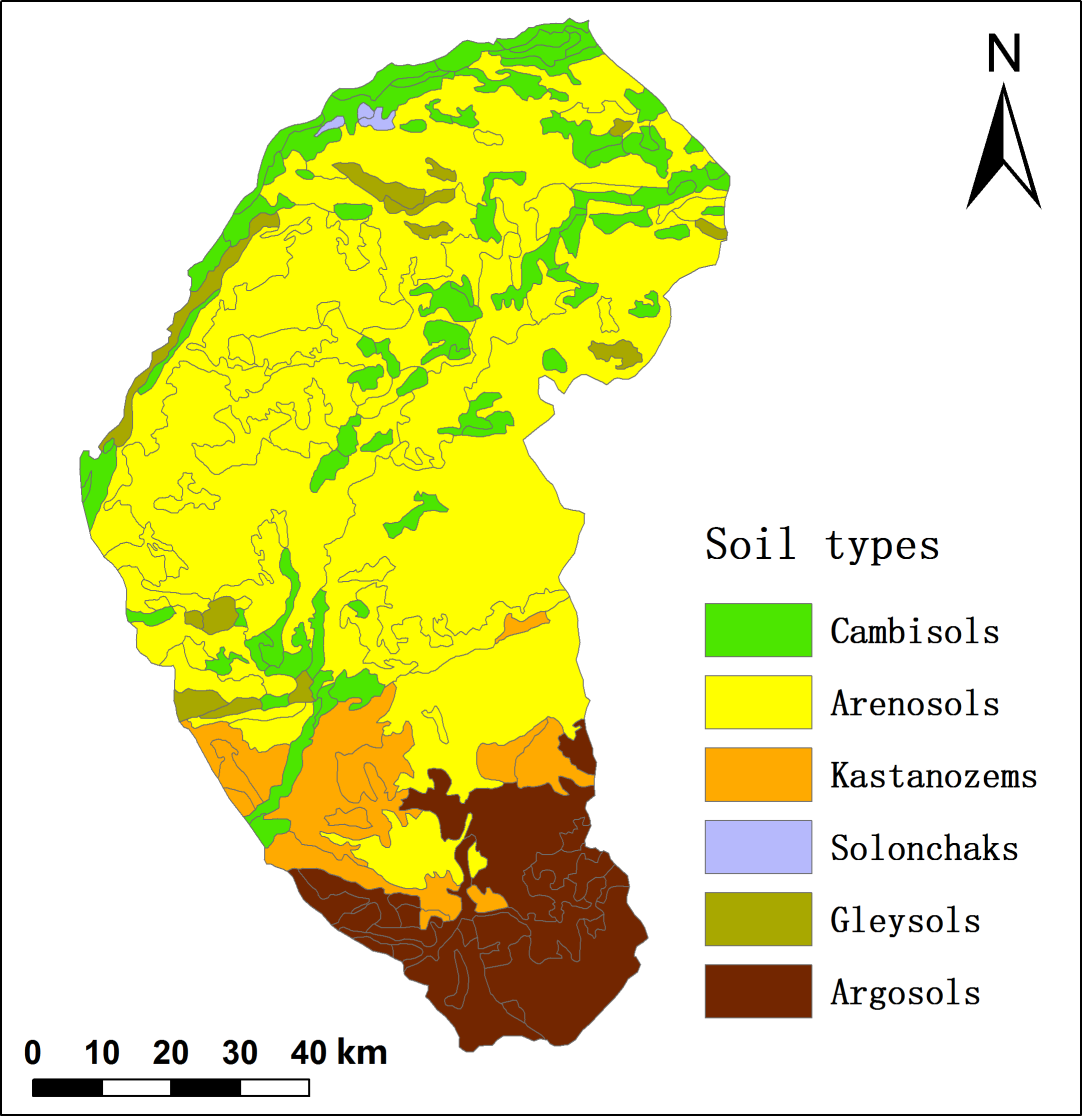
The distribution of soil types in Naiman banner. **Reprinted from [30] under a CC BY license, with permission from [Wang Guoguang], original copyright [1984].** There are six soil types in Naiman Banner according to the second national soil survey: Arenosols cover 58.2% of the total area, Cambisols cover 20.0%, Argosols cover 12.1%, Kastanozems cover 8.4%, Gleysols cover 1.2%, and Solonchaks cover 0.1%.

Naiman Banner has a temperate continental semiarid monsoon climate regime. The mean annual precipitation is 366 mm, of which 70% falls from June to August, and the mean annual potential evaporation is 1935 mm. The average annual air temperature ranges from 6.0 to 6.5°C, with a minimum monthly mean of –13.2°C in January and a maximum monthly mean of 23.5°C in July. The frost-free period ranges from 130 to 150 d. The mean wind speed is 4.3 m·s^-1^. Fig 2 shows the current land-use and cover types: the main types are cropland (34.7% of the area), grassland (31.4%), woodland (10.4%), and sandy land (19.2%). Sandy land was mainly distributed in the central region. The zonal soils are classified as Kastanozems, but as a result of desertification, the current dominant soils are Arenosols with a coarse texture and a loose structure [28]. There are six soil types in Naiman Banner according to the second national soil survey (Fig 3): Arenosols covers 58.2% of the total area, Cambisols covers 20.0%, Argosols covers 12.1%, Kastanozems covers 8.4%, Gleysols covers 1.2%, and Solonchaks cover 0.1% [23].

### Soil sampling

In this study, research is permitted by Northwest Institute of Eco-Environment and Resources, Chinese Academy of Sciences. Thus, our field study didn’t require specific permission from managers of these areas. However, we confirmed that all samples collected on private land were obtained with permission from the land owner. We collected the SOC and TN data as part of a regional soil investigation. Samples to a depth of 100 cm were obtained at 332 sites in Naiman Banner during July and August 2011, using a 10 m × 10 m plot established at each of the 354 locations. The samples were collected at 15 randomly selected sampling points within each plot using a soil auger (2.5 cm in diameter) and divided into five layers: 0 to 10, 10 to 20, 20 to 40, 40 to 60, and 60 to 100 cm. A composite soil sample was prepared for each layer. Therefore, within each of the plots, we obtained five composite soil samples to a depth of 100 cm. To determine the soil bulk density, we selected three additional sampling points (replicates) in each plot and used a soil auger equipped with a stainless steel cylinder (5.5 cm in diameter and 4.2 cm in height) to obtain intact soil cores. The soil was sampled in 5-cm increments and the bulk density was calculated as the average of the resulting two to eight samples for each of the five soil layers; that is, the sample size ranged from two 5-cm samples for the 10-cm layers to eight 5-cm samples for the 40-cm layer [16].

### Laboratory analyses

We used a 2-mm mesh to remove roots and other large debris after air-drying the soil samples for 3 days. A portion of each soil sample was then ground to pass through a 0.25-mm mesh before determination of the C and N concentrations. A subsample of the air-dried soil was weighed and dried at 105°C for 24 h to determine the gravimetric water content. The SOC content was determined by the Walkley-Black dichromate oxidation procedure [31]. The TN content was determined using the Kjeldahl procedure [32].

### Statistical and geostatistical methods

We estimated the SOC and TN densities (ES, in kg·m^-2^) in the soil to a depth of 100 cm based on the soil bulk density (BD, in g·cm^-3^) and the elemental content (EC, in g·kg^-1^):

$$ES=\sum_{i=1}^{5}(EC\times BD\times T_{i})$$ (1)

Where *i* represents the soil layer (*i* = 1, 2, …, 5) and T_*i*_ represents the thickness of that layer (cm).

We calculated the mean, standard error (SE), variance, coefficient of variation (CV), and the maximum (Max) and minimum (Min) values as indicators of the central tendency and variation of the data.

The SOC and TN densities were divided into five subsets based on the dominant soil types in Naiman Banner: Gleysols, Cambisols, Kastanozems, Argosols, and Arenosols. Considering the regional landscape diversity, the SOC and TN densities were further divided into three sections (for the alluvial plains, sandy land, and low hills in Fig 1C and 1D), then the data was further grouped based on the three dominant land uses in the study area (cropland, woodland, and grassland) in each section. The SOC and TN densities were analyzed by land-use and soil types using one-way ANOVA. Where ANOVA revealed a significant difference, we used least-significant-difference (LSD) test to identify significant differences between pairs of land uses and pairs of soil types. The statistical tests were conducted using Microsoft Excel 2007 and SPSS 17.0.

Kriging is a geostatistical method that is commonly used to interpolate soil property datasets from discrete points to a spatially continuous surface [33, 34]. A vitally important spatial interpolation method named Regression kriging has the advantage of its ability to extend the method to a broad range of regression techniques and to allow separate interpretation of the two interpolated components [35].

The semi-variograms were used to quantify the spatial variation of each regionalized geostatistical variable. The semi-variogram *r (h)* represents half of the statistically expected squared difference between paired data values *z (x)* and *z (x + h)* with a lag distance *h*, and the results can be used to separate different locations [36]. The calculation is as follows:

$$r(h)=\frac{1}{2}E{\left[z(x)-z(x+h)\right]}^{2}$$ (2)

Where *E* represents the statistical expectation. The kriging weights are determined by the variogram and the configuration of the data. It is an optimal interpolator in the sense that the estimates are unbiased and have known minimum variances. Since the estimated variances can be determined and mapped like the estimates, confidence intervals can be calculated and placed in the estimates for a given statistical distribution. Different statistical distributions can be compared to determine which one best fits the data. There are many examples of mapping soil properties in this way [37–39]. The semi-variogram model can be used to quantify spatial autocorrelation and provide input parameters for spatial interpolation [40]. Because of the basic geographic principle that things that are closer together tend to be more alike, measured points that are close will generally have a smaller squared difference than those that are farther apart. Once each pair of locations has been binned and plotted, a model can be fit through the bins. The range, sill, and nugget of the distribution are commonly used to describe these models.

## Results and discussion

### Descriptive statistics

Table 1 summarizes the basic statistical parameters for our dataset for the three land uses (cropland, woodland, and grassland) and five soil types (Cambisols, Arenosols, Kastanozems, Gleysols, and Argosols). The SOC density for the overall study area ranged from 0.37 to 13.74 kg·m^-2^, with a mean of 4.85 kg·m^-2^. The TN density ranged from 0.03 to 1.63 kg·m^-2^, with a mean of 0.54 kg·m^-2^. The corresponding CVs were 67.4 and 66.9%. Both SOC and TN had a relatively high CV, probably due the heterogeneity of the land use patterns, soil types, and other factors. The C/N ratio in the different soil types decreased in the following order: Cambisols (9.41) > Arenosols (9.17) > Argosols (9.10) > Gleysols (8.88) > Kastanozems (8.47). In the different land uses, it decreased as follows: woodland (9.61) > grassland (9.41) > cropland (9.02).

**Table 1:** Statistical characteristics of the soil organic carbon (SOC) and total nitrogen (TN) densities (kg m^-2^ to a depth of 100 cm) in Naiman Banner of northeastern China (n = 332 sample sites).

Category	n	Parameter	Mean	Min	Max	S.E
**Overall**	**332**	SOC	4.85	0.37	13.74	0.18
		TN	1.63	0.03	1.63	0.02
**Cropland**	**96**	SOC	5.12	1.11	13.48	0.29
		TN	0.57	0.09	1.35	0.03
		C/N	9.02	6.34	13.76	0.13
**Woodland**	**71**	SOC	3.54	0.60	8.14	0.17
		TN	0.38	0.03	0.86	0.02
		C/N	9.61	4.92	17.48	0.26
**Grassland**	**25**	SOC	3.93	1.58	10.17	0.41
		TN	0.42	0.15	1.03	0.04
		C/N	9.41	7.01	12.69	0.26
**Cambisols**	**39**	SOC	5.04	1.11	12.40	0.50
		TN	0.53	0.12	1.35	0.05
		C/N	9.41	7.81	12.69	0.14
**Arenosols**	**121**	SOC	3.20	0.37	13.12	0.22
		TN	0.35	0.03	1.30	0.02
		C/N	9.17	4.73	17.48	0.17
**Kastanozems**	**26**	SOC	3.92	2.44	8.14	0.24
		TN	0.46	0.29	0.81	0.02
		C/N	8.47	6.34	11.97	0.26
**Gleysols**	**82**	SOC	7.85	1.09	13.74	0.41
		TN	0.89	0.12	1.63	0.05
		C/N	8.88	5.01	12.51	0.13
**Argolsols**	**64**	SOC	4.41	1.18	11.49	0.23
		TN	0.49	0.08	1.30	0.02
		C/N	9.10	4.92	15.09	0.20

### Effects of soil type and land use on the SOC and TN densities

**Fig 4:**
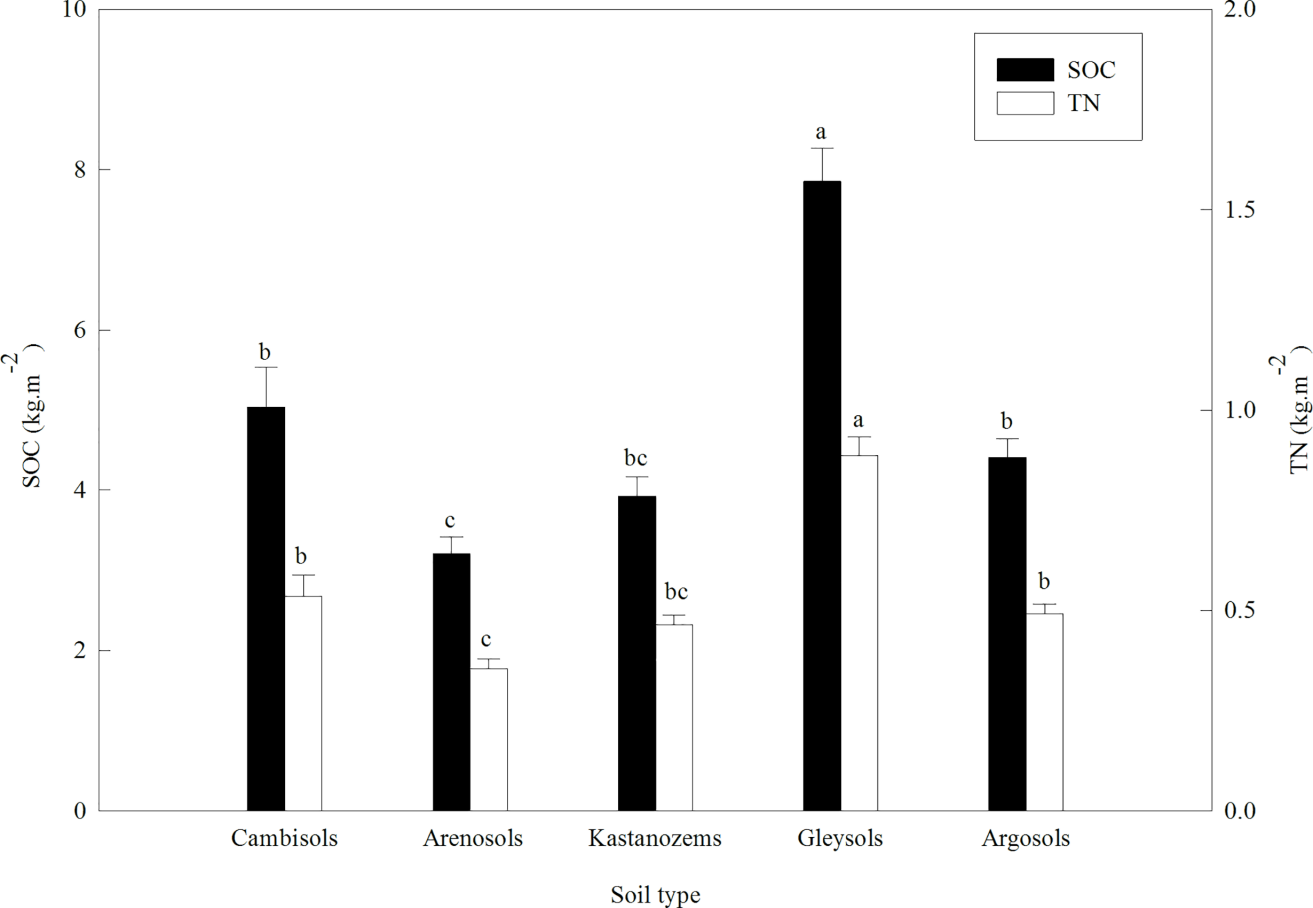
Differences in the soil organic carbon (SOC) and total nitrogen (TN) densities to a depth of 100 cm among the five soil types. For a given element, bars labeled with different letters differ significantly (one-way ANOVA followed by LSD test, P < 0.01). Values are means ± SE.

To explore the relationships between the soil nutrients (SOC and TN) and the soil types, we compared SOC and TN among the soil types. We calculated that the skewness of the distributions for the SOC and TN densities were 0.68 and 0.73, respectively, and that the corresponding kurtosis values were –0.56 and –0.35; since their absolute values are less than 1, LSD tests can be applied to the data. In our study area, the SOC and TN densities were both in the following order: Gleysols > Cambisols > Argosols > Kastanozems > Arenosols. Fig 4 summarizes the SOC and TN densities for the five soil types, and shows significant differences between Cambisols, Arenosols, and Gleysols; between Arenosols and both Gleysols and Argosols; between Kastanozems and Gleysols; and between Gleysols and Argosols.

These results may have several explanations. The Gleysols soils tend to accumulate organic matter (thus, SOC and TN) because the waterlogged conditions slow its decomposition into more labile forms [41]. In addition, the adsorption of humic substances is accelerated due to Cambisols is rich in Ca^2+^, leading to the increasing of SOC and TN accumulation rate [42]. In contrast, the low vegetation cover at sites with Arenosols means that there are low inputs of litter; this combines with increased water and wind erosion, which decrease the SOC and TN densities to values lower than those in the other soil types [43, 44]. The Argosols and Kastanozems have intermediate characteristics, and therefore have intermediate SOC and TN densities.

**Fig 5:**
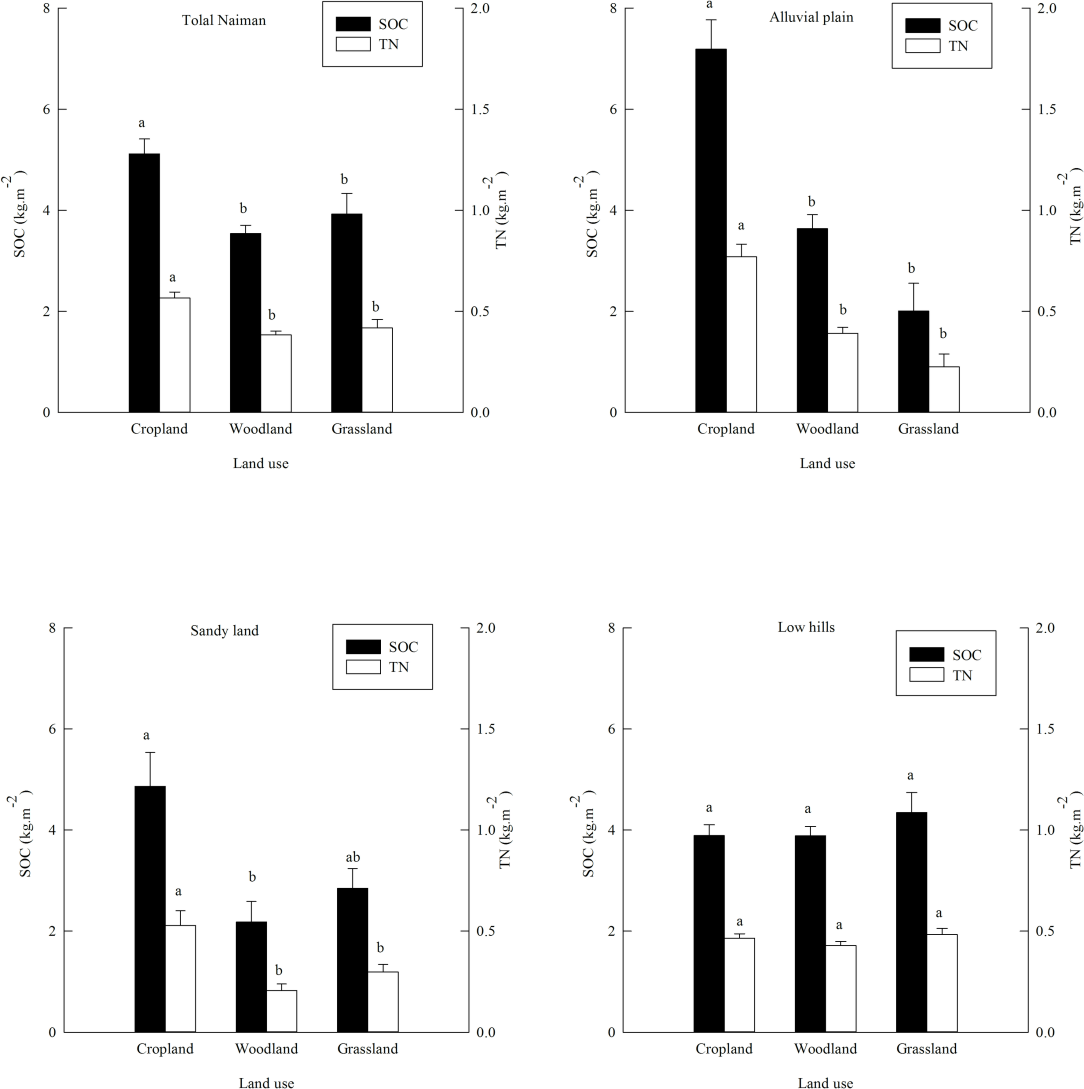
Differences in the soil organic carbon (SOC) and total nitrogen (TN) densities to a depth of 100 cm for all sites combined in Naiman Banner and for the three land use types. For a given parameter, bars labeled with different letters differ significantly (P < 0.05, one-way ANOVA followed by LSD test). Values are means ± SE.

Fig 5 shows the SOC and TN densities for the different land uses. For all sites combined in Naiman Banner and for sites in the alluvial plains, the SOC and TN densities of cropland were significantly greater than those of woodland and grassland, which did not differ significantly for either parameter, but two parameters of woodland were higher than grassland in alluvial plain. For the sites in sandy land, cropland again had the highest SOC and TN densities; SOC was significantly higher than in woodland but not grassland, and TN was significantly higher than both other types, two parameters of grassland were higher than woodland, but woodland and grassland did not differ significantly for either parameter. For sites in the low hills, though neither SOC nor TN density differed significantly among the land use types, the SOC and TN densities of grassland were slightly higher than those of woodland and cropland. In a sense, to sequester carbon and nitrogen in the soil, the conversion of degraded land into woodland rather than grassland should be advocated in alluvial plains, whereas more grassland should be established in sandy land and low hills.

In our study, the SOC and TN densities of cropland were higher than those of woodland and grassland in most cases. This differs somewhat from the results of previous studies [45–47], in which grassland or forest generally had significantly higher SOC and TN storage than cropland. Owing to that afforested grassland and woodland sites had experienced severe soil impoverishment that has only recently been mitigated by the restoration programs. In contrast, the current cropland was mostly established from native natural grasslands that had a high initial soil organic matter density [16]. SOC was likely increased due to the fertilizer addition and irrigation which increases SOC by increasing plant growth and litterfall, and by the addition of manure, which directly increases SOC since that the manure contains large amounts of organic carbon and other nutrients, including organic nitrogen [48–51].

### Effects of SOC on TN and BD

**Fig 6:**
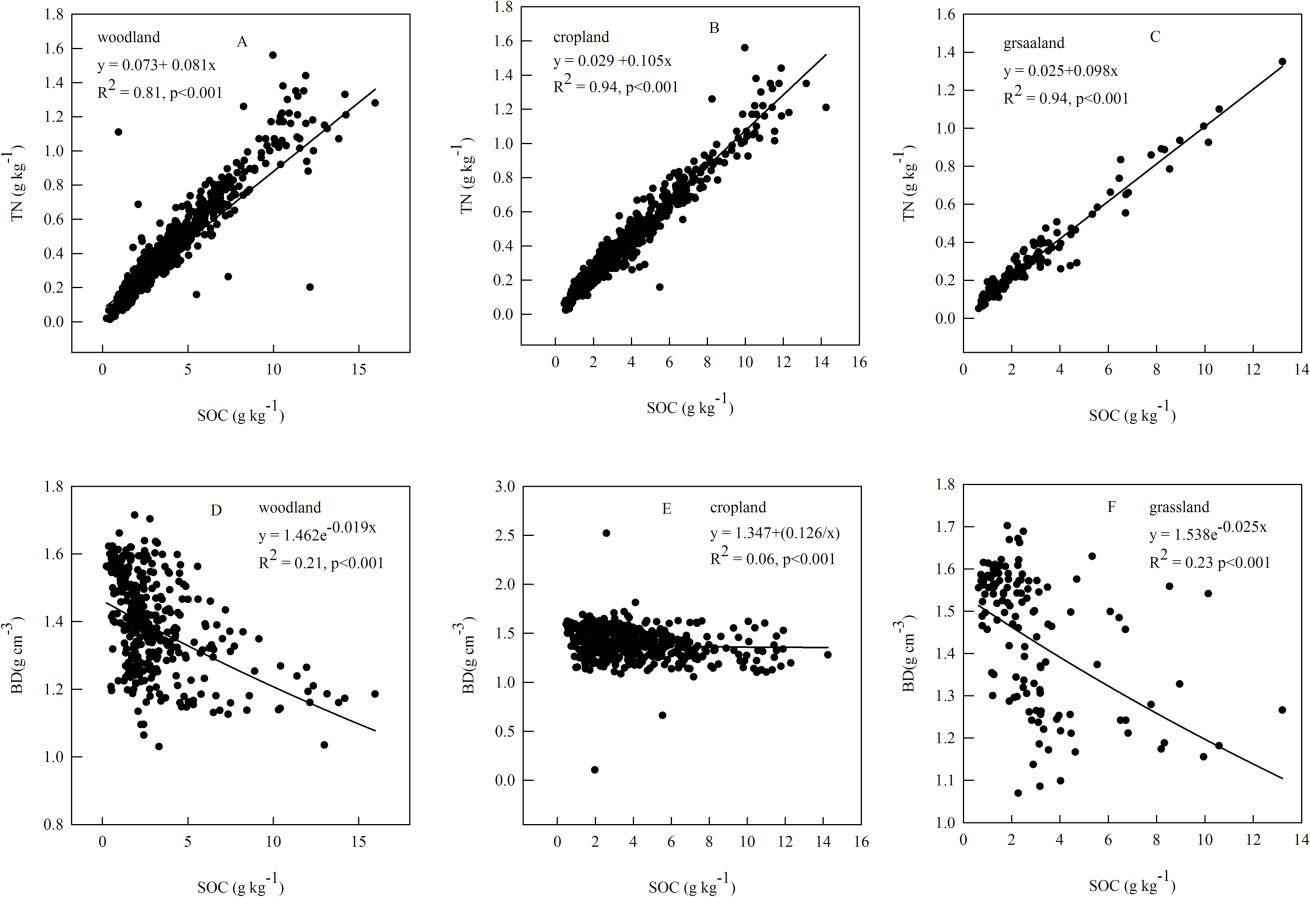
Regression equations for the relationship between (A, B, C) the soil organic carbon (SOC) content and the total nitrogen (TN) content and (D, E, F) between the bulk density (*BD*) and SOC for the three dominant land uses in Naiman Banner.

As expected, the TN content was strongly and significantly positively correlated with the SOC content for all three land uses (woodland, cropland, and grassland) in the study area (Fig 6A–6C). Similarly as expected, BD was significantly negatively correlated with SOC content. For woodland (Fig 6A and 6D), TN (*R*^*2*^ *= 0*.*81*, *p < 0*.*001*) increased with increasing SOC content, but BD (*R*^*2*^ *= 0*.*21*, *p < 0*.*001*) decreased with increasing SOC content. For cropland (Fig 6B and 6E), TN (*R*^*2*^ *= 0*.*94*, *p < 0*.*001*) increased with increasing SOC content, but BD (*R*^*2*^ *= 0*.*06*, *p < 0*.*001*) decreased with increasing SOC content. For grassland (Fig 6C and 6F), treads were similar to those for woodland; that is, BD (*R*^*2*^ *= 0*.*23*, *p < 0*.*001*) decreased with increasing SOC content, but TN (*R*^*2*^ *= 0*.*94*, *p < 0*.*001*) increased with increasing SOC content. In general, the soils with the lowest BD had the highest organic matter density, which agrees with previous research [52]. In general, SOC concentration will be significantly reduced due to the C mineralization, which is the conversion from the organic C form to inorganic compound as a result of decomposition reactions [53]. However, it will slower the process of C mineralization in the nitrogen-rich soil [54], leading to a high SOC concentration.

### Vertical distribution of SOC and TN content in the different soil types

**Fig 7:**
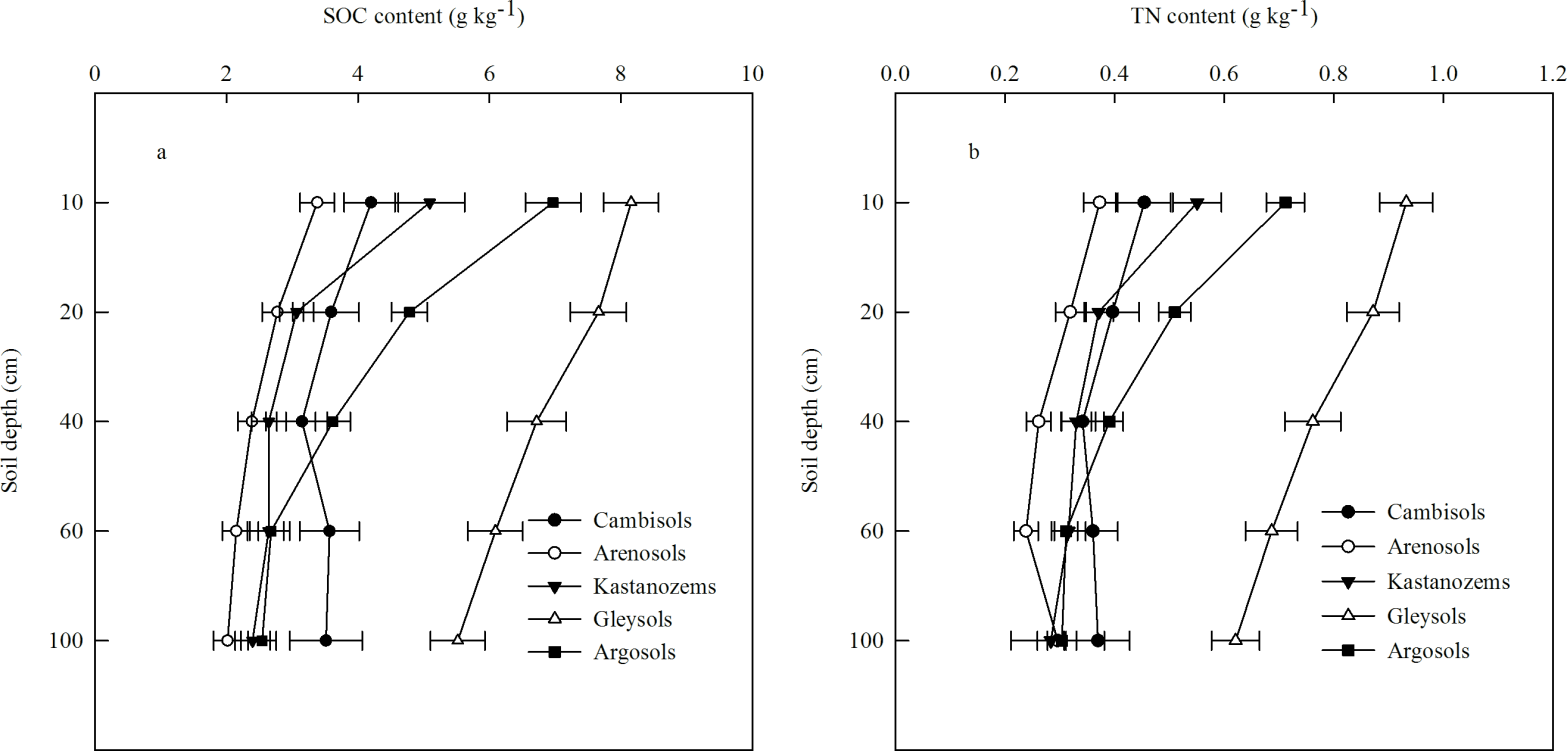
**Vertical distribution of the (a) soil organic carbon (SOC) density and (b) total nitrogen (TN) density to a depth of 100 cm. Data points were plotted at the bottom of each soil layer.** Values are means ± SE.

We also compared the vertical distributions of the SOC and TN contents among the five soil types (Fig 7). In most cases, the SOC and TN contents both decreased with increasing soil depth. The SOC content decreased most rapidly with increasing depth in the Gleysols and Argosols, and most slowly in the Arenosols. The root distribution may partially explain this trend, since the roots are an important source of SOC (both through root exudates and through root mortality). For example, the woodland and had plants with the deepest roots (i.e., trees), and therefore showed the slowest decrease in SOC with increasing depth; this can be seen in the slow SOC change in the Arenosols, which are the dominant soils of woodland and deep- rooted shrubs (50.56%). In contrast, SOC decreased relatively rapidly with increasing depth in the Cambisols, which are the dominant soils for shallow-rooted grasses and crops (55.26%). In addition, the allocation of photosynthate to aboveground and belowground biomass would have affected the relative amount of C that eventually fell to the soil surface as litter [55]. This can be seen in the Argosols, which were common in woodland areas (41.67%); as a result, the relatively high inputs of leaf litter from the trees led to high SOC near the soil surface.

### Analysis of the spatial variation of SOC and TN

Table 2 summarizes the parameters of three semi-variogram models that we tested (Spherical, Exponential, and Gaussian). The nugget/sill ratio for SOC and TN had the smallest values of three models (31.9 and 30.2%, respectively) in the exponential models, and their partial sill/sill ratios (68.1 and 69.8%, respectively) were larger than those of the other two models. Thus, we adopted the exponential models in this study because the strength of the spatial correlation was stronger with this model form. A variable is considered to have a strong spatial dependence if the nugget/sill ratio is less than 25%, and has a moderate spatial dependence if the ratio is between 25% and 75%; otherwise, the variable has a weak spatial dependence. On this basis, SOC and TN both showed moderately strong spatial dependence using the exponential model.

**Table 2:** Parameters of the variogram models for the soil organic carbon (SOC) and total nitrogen (TN) densities to a depth of 100 cm in Naiman Banner.

	Model for SOC			Model for TN		
	Spherical	Exponential	Gaussian	Spherical	Exponential	Gaussian
**Range (m)**	49417.68	55967.63	41594.67	40496.23	58837.75	34741.93
**Nugget**	0.28	0.23	0.33	0.25	0.21	0.3
**Partial sill**	0.43	0.49	0.39	0.41	0.5	0.36
**Sill**	0.71	0.72	0.71	0.66	0.71	0.66
**Nugget/sill**	38.84%	31.89%	45.72%	37.60%	30.18%	45.69%
**Partial sill /sill**	61.16%	68.11%	54.28%	62.40%	69.82%	54.31%

### Spatial distribution of SOC and TN densities

**Fig 8:**
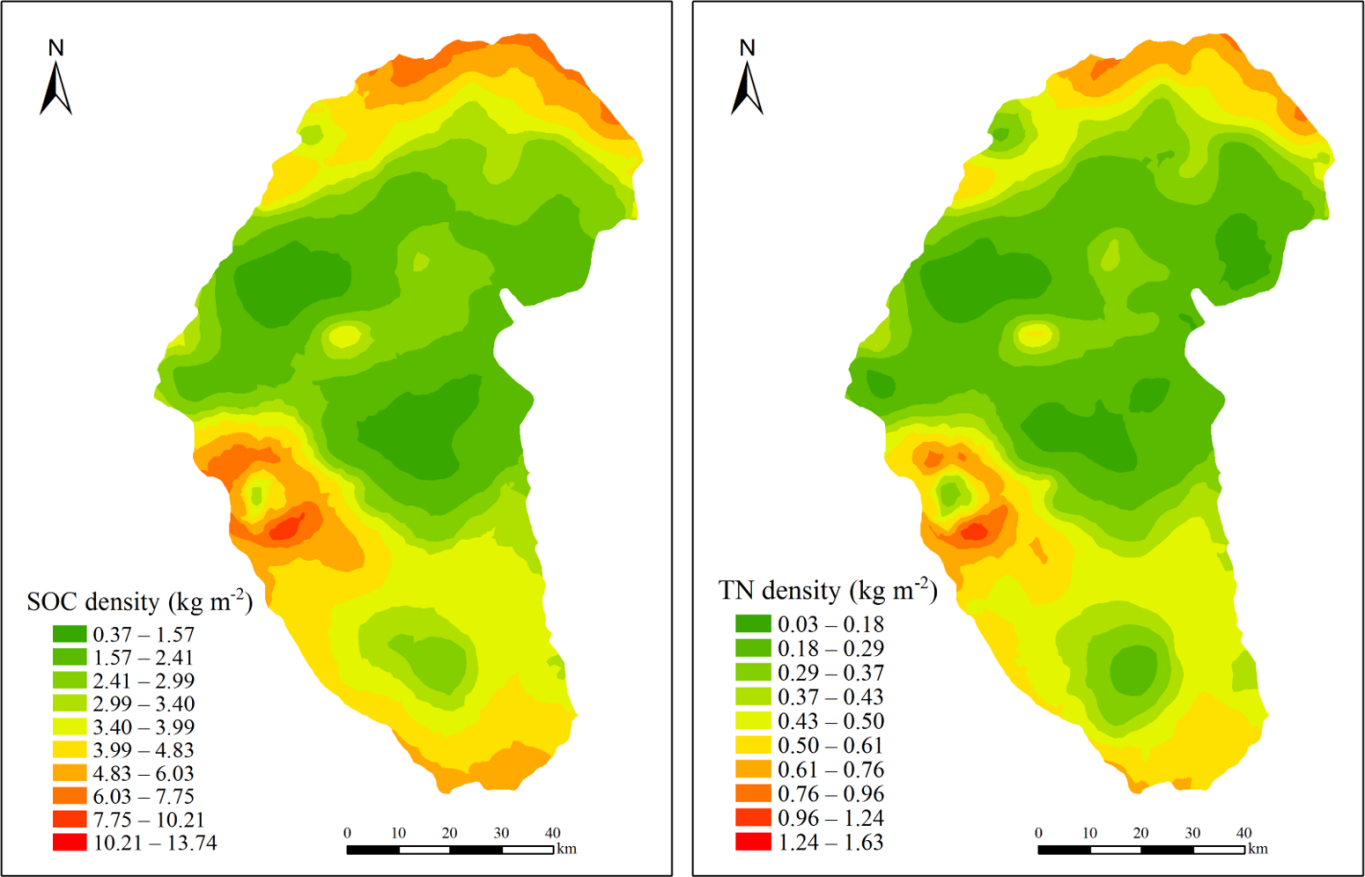
Interpolated distributions (by simple kriging) of the soil organic carbon (SOC) and total nitrogen (TN) densities to a depth of 100 cm in Naiman Banner.

Fig 8 shows the results of simple kriging interpolation for the SOC and TN densities. Overall, moving from north to south, the SOC and TN densities decreased to reach a minimum near the center-north part of Naiman Banner, and then increased.

For SOC, the interpolated values ranged from 0.37 to 13.74 kg·m^-2^. The sites with the highest SOC density were mainly located in the southern area, which belonged to the Source Region of the West Liaohe River protected area. There was also a relatively high concentration at the northern edge of the study area, in an area of alluvial plains. On the other hand, SOC density was lowest in the central and northern region, which were predominantly sandy land that was part of the Horqin Sandy Land protected area. Another area with obviously high SOC values was located in the western part of Naiman Banner, in the Source Region of the West Liaohe River protected area. There was also a large area with unusually low SOC and TN densities in the center of the southern region, in low hills with cropland and Kastanozems. Both the vegetation type and the soil type in this area tend to have low nutrient contents (Table 1), and the combination explains the low values in this area.

For TN, the density ranged from 0.03 to 1.63 kg·m^-2^. The spatial distribution of TN density was very similar to that of SOC. TN density was lowest in the central and northern regions, and highest in the southern region. This agrees with the analysis in Fig 6, which shows a strong and significant linear relationship between the SOC and TN densities.

Table 3 summarizes the mean SOC and TN densities for the three sections (alluvial plains, sandy land, and low hills) in Naiman Banner. The average SOC and TN densities were highest in the low hills, followed by the alluvial plains, and were lowest in the sandy land; neither parameter differed significantly between alluvial land and low hills, but values of both parameters were significantly lower in the sandy land. These results can be attributed to differences in the soil types and ecological functions in the three sections. The most common soils in the alluvial plains were Cambisols, Kastanozems and Argosols in the low hills and Arenosols in the sandy land. Firstly, Cambisols sequester large amounts of SOC and TN [42], whereas Arenosols are barren with low nutrient content. Sandy land was located primarily in the Horqin Sandy Land area that was protected for sand fixation, strong wind erosion led to low vegetation cover in this area, leading to lower densities of SOC and TN [44]. In contrast, low hills belonged to the Source Region of the West Liaohe River area that was protected for water conservation, thus low hills were common and had plenty of water and lush grasses; as a result, they had the highest SOC and TN densities in Naiman Banner.

**Table 3:** Mean values of the soil organic carbon (SOC) and total nitrogen (TN) densities to a depth of 100 cm in the alluvial plains, sandy land, and low hills of Naiman Banner.

	Mean density (kg m^-2^)		
	Alluvial plains	Sandy land	Low hills
**SOC**	4.96 ± 0.45a	2.92 ± 0.28b	5.43 ± 0.24a
**TN**	0.53 ± 0.05a	0.31 ± 0.03b	0.62 ± 0.03a

For a given parameter, values labeled with different letters differ significantly (P < 0.05; one-way ANOVA, followed by LSD test).

## Conclusions

Our results clearly indicated that both soil type and land use significantly affected the SOC and TN densities. SOC and TN densities were highest for the Gleysols, and lowest for the Arenosols. Differences in SOC and TN densities among the soil types were generally significant, except for similar values in the Argosols and Kastanozems. The effect of land use on the SOC and TN densities differed significantly among the primary land uses, in part because of differences in the dominant soil types. The SOC and TN densities of cropland were significantly greater than those of woodland and grassland (all soil types combined). Neither the SOC density nor the TN density differed significantly among the different land uses in the low hills. Our results suggest that there is considerable potential to sequester carbon and nitrogen in the soil via the conversion of desertified sandy land into woodland rather than grassland in alluvial plains, whereas more grassland should be established in sandy land and low hills. In addition, SOC and TN contents decreased significantly with increasing BD. From north to south, SOC and TN densities both decreased initially and then increased, with the lowest values in the sandy land towards the center of Naiman Banner. These trends can be mostly attributed to the different soil types and land uses of these areas.

## Supporting information

S1 Fig The granted permission to the Fig 1. (JPG)

S2 Fig The granted permission to the Fig 2. (JPG)

S3 Fig The granted permission to the Fig 3. (JPG)

S4 Fig The granted permission to the Fig 8. (JPG)

S1 Table Soil organic carbon (SOC) and total nitrogen (TN) contents in the soil to a depth of 100 cm. (XLSX)

S2 Table Soil bulk density. (XLSX)

S3 Table SOC and TN densities in the soil to a depth of 100 cm. (XLSX)

S4 Table Differences in the SOC and TN densities to a depth of 100 cm among the five soil types. (XLSX)

S5 Table Differences in the SOC and TN densities to a depth of 100 cm for all sites combined in Naiman Banner and for the three land use types. (XLSX)

S6 Table Regression equations for the relationship between the SOC content and the TN content and between the BD and SOC for the three dominant land uses in Naiman Banner. (XLSX)
