# Supplementary figures and images for: Spatial pattern of soil organic carbon and total nitrogen, and analysis of related factors in an agro-pastoral zone in Northern China
Source: PLoS One. 2018 May 17;13(5):e0197451. doi: 10.1371/journal.pone.0197451 (PMC5957344; doi:10.1371/journal.pone.0197451)

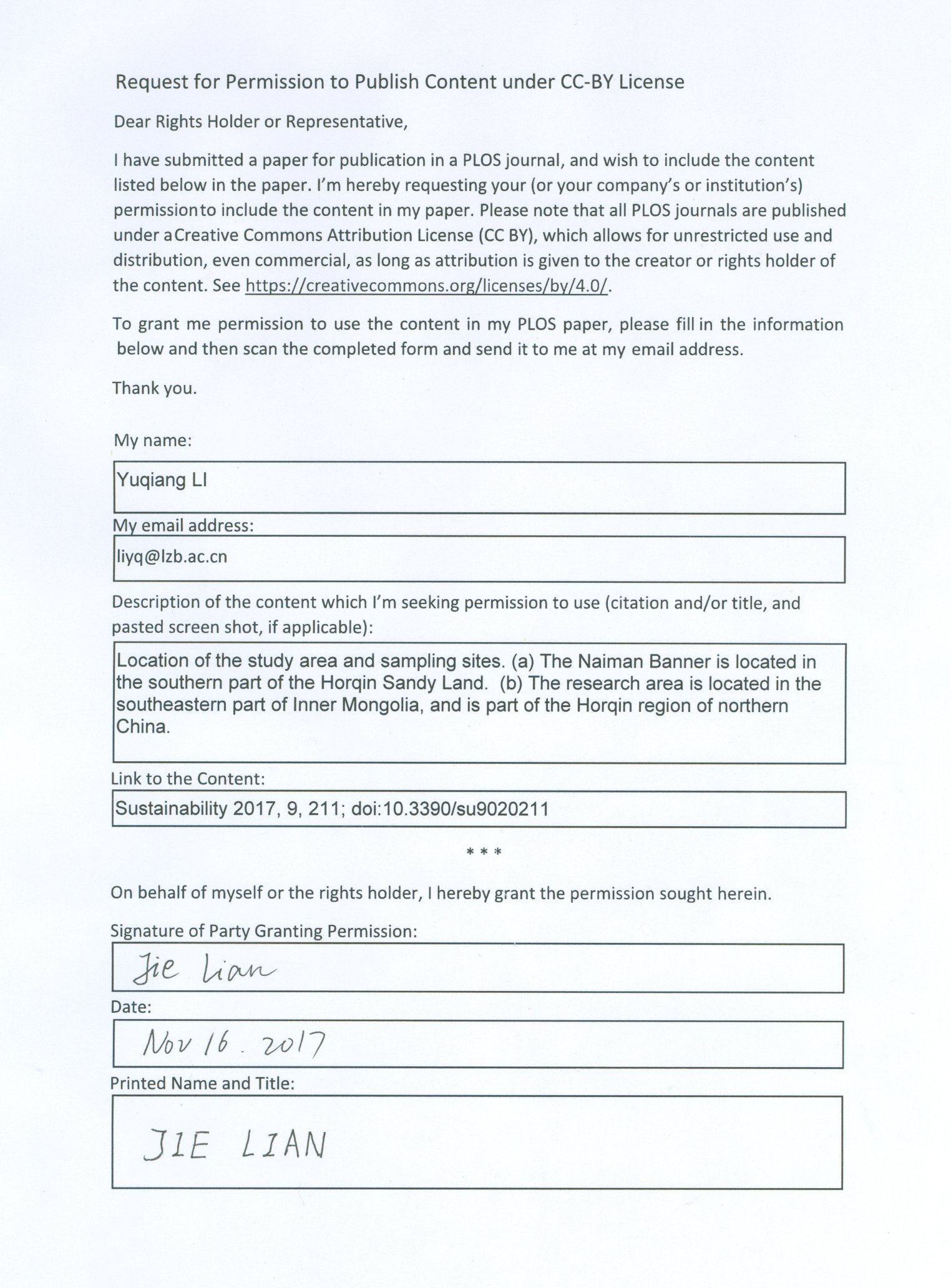

Supplement: S1 Fig — (JPG) [file pone.0197451.s001.jpg]

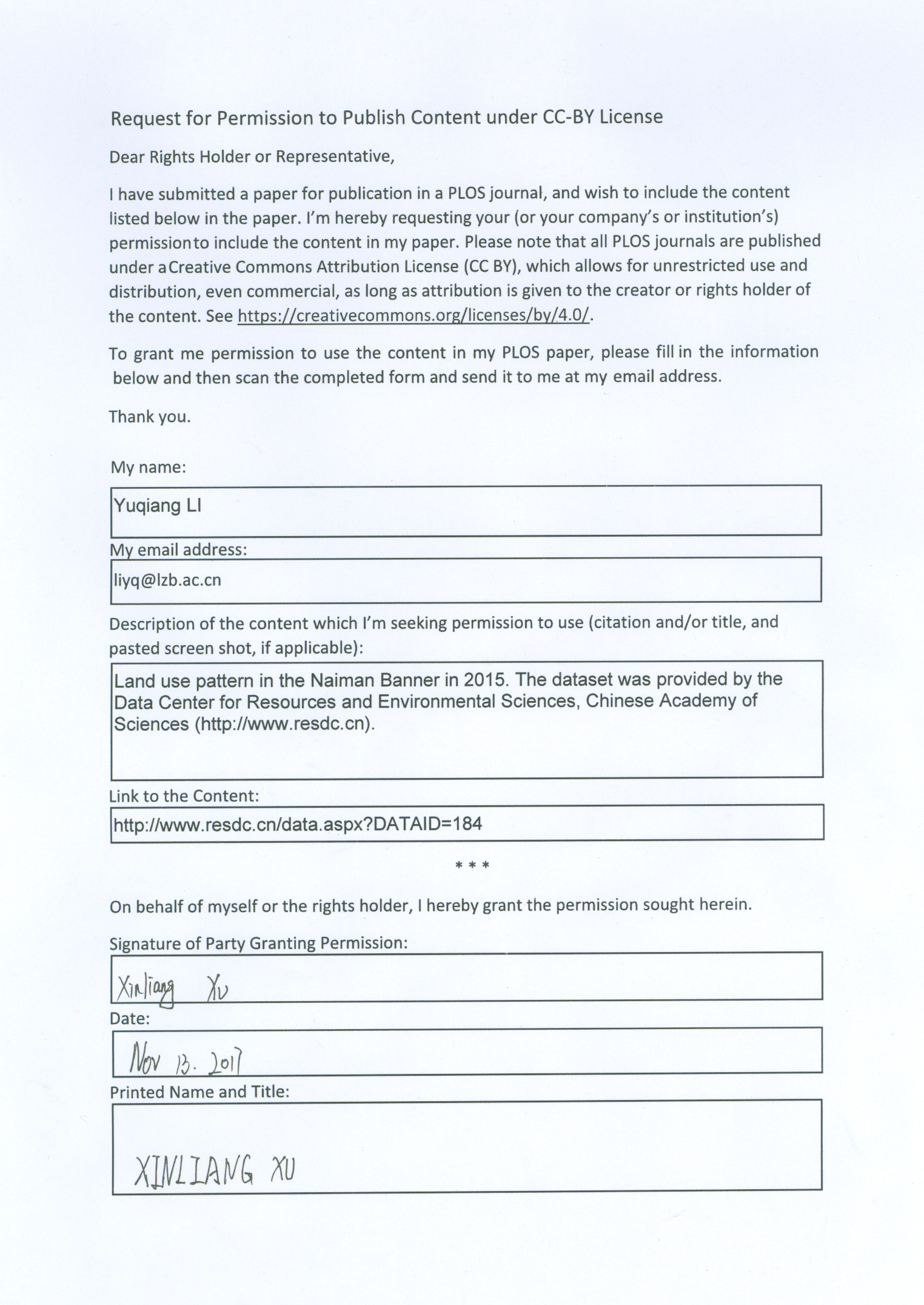

Supplement: S2 Fig — (JPG) [file pone.0197451.s002.jpg]

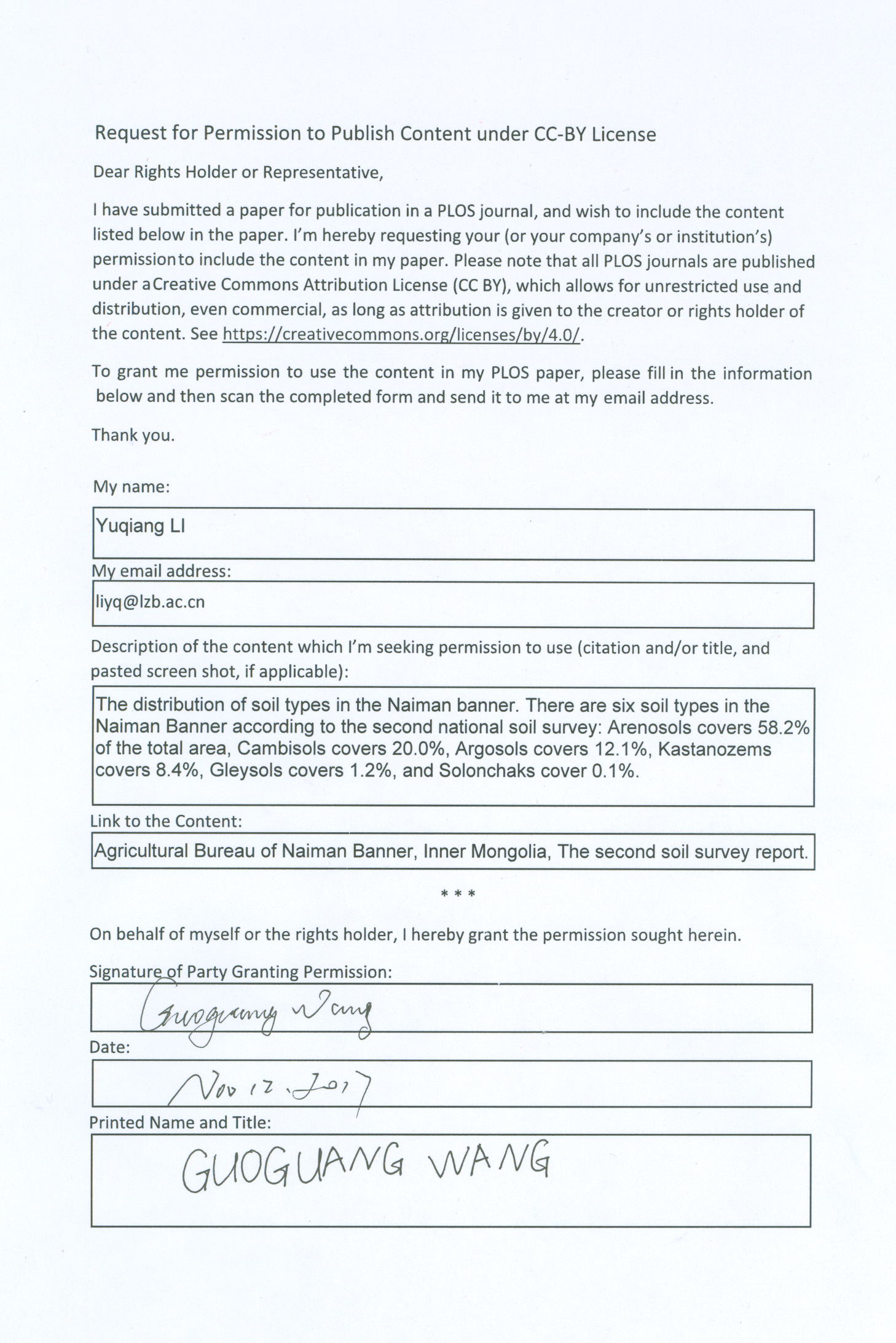

Supplement: S3 Fig — (JPG) [file pone.0197451.s003.jpg]

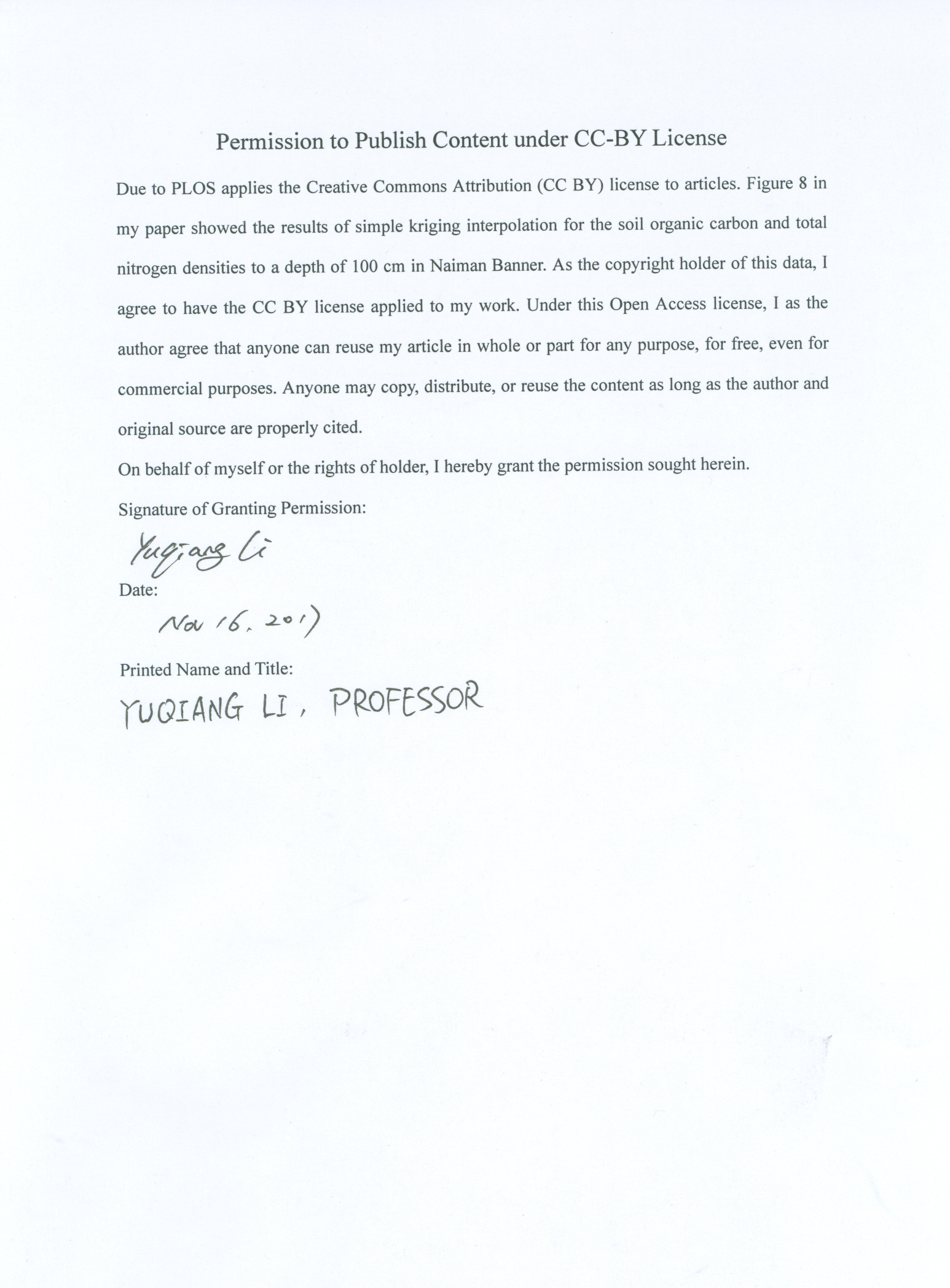

Supplement: S4 Fig — (JPG) [file pone.0197451.s004.jpg]
